# Traditional Chinese medicine bufalin inhibits infectious hematopoietic necrosis virus infection *in vitro* and *in vivo*

**DOI:** 10.1128/spectrum.05016-22

**Published:** 2024-01-30

**Authors:** Jing-Zhuang Zhao, Li-Ming Xu, Lin-Fang Li, Guang-Ming Ren, Yi-Zhi Shao, Qi Liu, Tong-Yan Lu

**Affiliations:** 1 Heilongjiang River Fisheries Research Institute, Chinese Academy of Fishery Sciences, Harbin, China; 2 Key Laboratory of Aquatic Animal Diseases and Immune Technology of Heilongjiang Province, Department of Aquatic Animal Diseases and Control, Harbin, China; USDA-ARS National Center for Cool and Cold Water Aquaculture, Kearneysville, West Virginia, USA

**Keywords:** infectious hematopoietic necrosis virus, rainbow trout, bufalin, traditional Chinese medicine, antiviral drugs

## Abstract

Infectious hematopoietic necrosis virus (IHNV) causes infectious hematopoietic necrosis and severe economic losses to salmon and trout aquaculture worldwide. Currently, the only commercial vaccine against IHNV is a DNA vaccine with some biosafety concerns. Hence, more effective vaccines and antiviral drugs are needed to prevent IHNV infection. In this study, 1,483 compounds were screened from a traditional Chinese medicine monomer library, and bufalin showed potential antiviral activity against IHNV. The 50% cytotoxic concentration of bufalin was >20 µM, and the 50% inhibitory concentration was 0.1223 µΜ against IHNV. Bufalin showed the inhibition of diverse IHNV strains *in vitro*, which confirmed that it had an inhibitory effect against all IHNV strains, rather than random activity against a single strain. The bufalin-mediated block of IHNV infection occurred at the viral attachment and RNA replication stages, but not internalization. Bufalin also inhibited IHNV infection *in vivo* and significantly increased the survival of rainbow trout compared with the mock drug-treated group, and this was confirmed by *in vivo* viral load monitoring. Our data showed that the anti-IHNV activity of bufalin was proportional to extracellular Na^+^ concentration and inversely proportional to extracellular K^+^ concentration, and bufalin may inhibit IHNV infection by targeting Na^+^/K^+^-ATPase. The *in vitro* and *in vivo* studies showed that bufalin significantly inhibited IHNV infection and may be a promising candidate drug against the disease in rainbow trout.

**IMPORTANCE:**

Infectious hematopoietic necrosis virus (IHNV) is the pathogen of infectious hematopoietic necrosis (IHN) which outbreak often causes huge economic losses and hampers the healthy development of salmon and trout farming. Currently, there is only one approved DNA vaccine for IHN worldwide, but it faces some biosafety problems. Hence, more effective vaccines and antiviral drugs are needed to prevent IHNV infection. In this study, we report that bufalin, a traditional Chinese medicine, shows potential antiviral activity against IHNV both *in vitro* and *in vivo*. The bufalin-mediated block of IHNV infection occurred at the viral attachment and RNA replication stages, but not internalization, and bufalin inhibited IHNV infection by targeting Na^+^/K^+^-ATPase. The *in vitro* and *in vivo* studies showed that bufalin significantly inhibited IHNV infection and may be a promising candidate drug against the disease in rainbow trout.

## INTRODUCTION

Infectious hematopoietic necrosis (IHN) is an important disease of salmon and trout that has caused huge economic losses to the global fish farming industry (1). IHN was first reported in sockeye salmon (*Oncorhynchus nerka*) farms in Washington and Oregon in the 1950s, but the disease did not spread widely for the following 20 years (2). In the early 1980s, IHN gradually spread to many countries around the world with the trade of fry and adult fish in countries including Japan (3), Iran (4), Canada (5), Korea (6), Russia (7), Netherlands (8), and China (9). An outbreak of IHN can cause >80% mortality depending on the fish species and size and even 100% mortality in fry (10, 11). Therefore, IHN is defined as a notifiable animal disease by the World Organization for Animal Health and many trading countries (11). The pathogen of IHN is infectious hematopoietic necrosis virus (IHNV) that belongs to the family *Rhabdoviridae* and the genus *Salmonid novirhabdovirus* (12). IHNV is a negative-sense, single-stranded RNA virus that encodes five structural proteins and a nonstructural protein in the order 3′-N-P-M-G-NV-L-5′; the N gene encodes nucleocapsid protein, P gene encodes phosphoprotein, M gene encodes matrix protein, G gene encodes glycoprotein, L gene encodes large polymerase, and NV gene encodes nonvirion protein (13). Phylogenetic analysis has shown that IHNV has evolved into five genogroups including U, M, L, E, and J (9). Although IHNV causes huge economic losses in salmon and trout farming, there is only one commercial vaccine worldwide, which was approved in 2005 for use in Canada (14). Hence, more effective vaccines and antiviral drugs are needed to prevent IHNV infection.

Cardiac glycosides (CGs) are secondary metabolites that are found widely in nature (15). CGs are divided into two subgroups depending on their source: the cardenolides are extracted from *Digitalis*, *Strophanthus,* and *Uregenia* plant species, and the bufadienolides are extracted from toad toxins (16–18). CGs were first used for the treatment of cardiovascular diseases (19) and research in the treatment of cancer, including breast (20), lung (21), pancreatic (22), and prostate (23) cancers. Recent studies of CGs revealed that they exhibited antiviral activity against *Retroviridae* (24, 25), *Filoviridae* (26), *Orthomyxoviridae* (27), *Herpesviridae* (28, 29), and *Coronaviridae* (30) families. The relationship between CGs and the *Rhabdoviridae* family has not been reported. Bufalin is a CG that is extracted from the parotid venom glands and skin epidermal glands of the Chinese toad *Bufo gargarizans*. In recent years, bufalin has been experimentally demonstrated to exhibit antiviral activity against hepatitis B virus, coronavirus, and vesicular stomatitis virus (31, 32).

Traditional Chinese medicine is a promising resource for the development of antiviral drugs. To identify antiviral candidates against IHNV, we screened 1,483 compounds from a traditional Chinese medicine monomer library. Bufalin is a CG that is extracted from toad toxins and exhibited a broad spectral antiviral activity against IHNV *in vitro* and *in vivo*. The anti-IHNV activity of bufalin was related to Na^+^/K^+^-ATPase activity, suggesting that Na^+^/K^+^-ATPase may be a potential therapeutic target for IHNV infection.

## RESULTS

### Screening of anti-IHNV drug from traditional Chinese medicine monomer library

**Fig 1:**
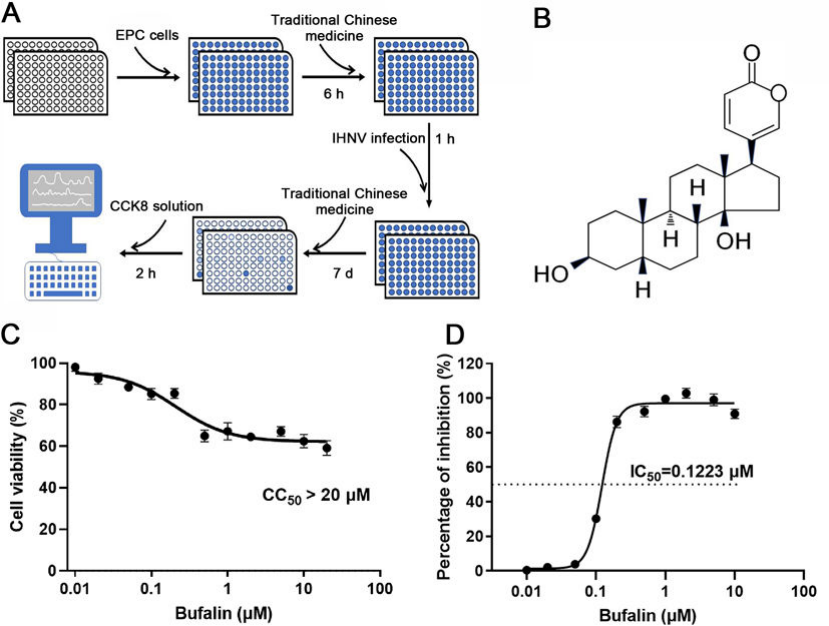
Screening of anti-IHNV drugs. (**A**) Schematic diagram of compound screening assay. (**B**) Chemical structure of bufalin. (**C**) Cytotoxicity of bufalin in EPC cells. (**D**) Inhibitory effect of bufalin against IHNV-Sn1203 in EPC cells.

To find novel compounds that inhibit IHNV infection, we screened ~1,500 small molecules from a traditional Chinese medicine monomer library in epithelioma *Papulosum cyprini* (EPC) cells using the IHNV-Sn1203 strain. Sn1203 was used in subsequent experiments unless specified otherwise. The anti-IHNV activity of different drugs was evaluated in EPC cells using the compound screening assay shown in Fig. 1A. Bufalin exhibited significant anti-IHNV activity, and its chemical structure is shown in Fig. 1B. To confirm the antiviral activity of bufalin, we performed a dose–response assay in EPC cells. The 50% cytotoxic concentration (CC_50_) of bufalin in EPC cells was >20 µM (Fig. 1C), and the 50% inhibitory concentration (IC_50_) was 0.1223 µΜ (Fig. 1D). Thus, the drug selectivity index (SI = CC_50_/IC_50_) of bufalin was >163.5. These results revealed that the anti-IHNV effect of bufalin was not caused by cell toxicity.

### Identification of bufalin as an effective inhibitor against IHNV-Sn1203

**Fig 2:**
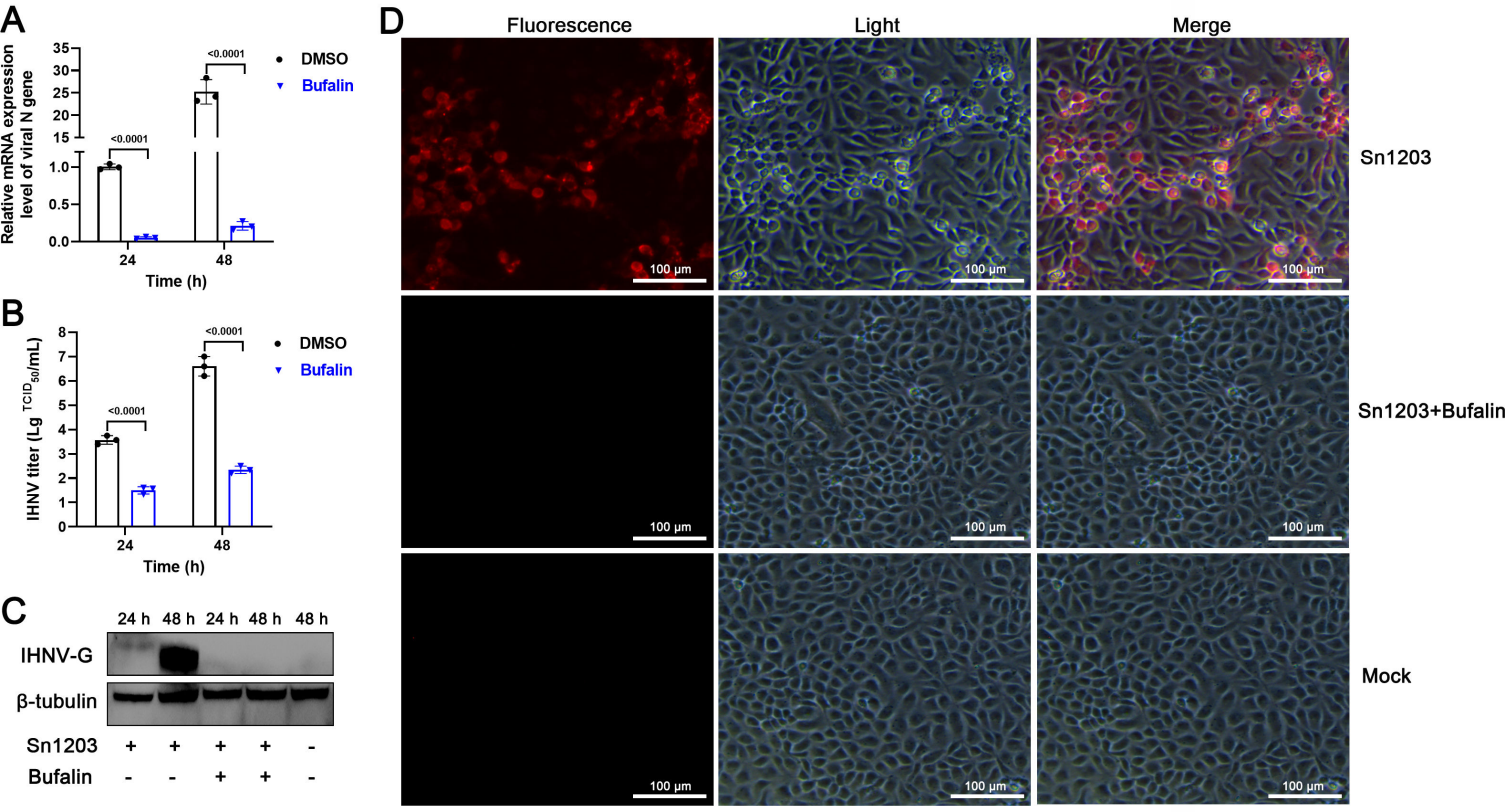
Antiviral effect of bufalin against IHNV-Sn1203 in EPC cells. (**A**) mRNA expression level of IHNV after bufalin treatment. (**B**) IHNV-Sn1203 titer after bufalin treatment. (**C**) Western blotting analysis of IHNV-Sn1203 G protein. (**D**) IFA detection of IHNV-Sn1203-infected cells. Red fluorescence represents IHNV-infected EPC cells in the field. The light image showed all the EPC cells in the field.

To further evaluate the antiviral ability of bufalin, we examined the viral replication after treatment of EPC cells with bufalin for 24 and 48 h. Reverse transcription–quantitative polymerase chain reaction (RT-qPCR) showed that intracellular viral replication was significantly inhibited by bufalin, and RNA expression was 19-fold lower at 24 h and 119-fold lower at 48 h compared with the DMSO control group (Fig. 2A). The extracellular viral titer was 2.1 log lower at 24 h and 4.3 log lower at 48 h, which was significantly lower compared with the DMSO control group (Fig. 2B). To examine the actual viral protein expression level, we performed western blotting and indirect immunofluorescence assay (IFA). Western blotting showed that IHNV-Sn1203 G protein expression was inhibited by bufalin at 24 and 48 h (Fig. 2C). IFA showed that bufalin significantly reduced the number of IHNV-Sn1203-infected cells compared with the mock-treated group (Fig. 2D). These results suggested that bufalin significantly inhibited IHNV-Sn1203 infection in EPC cells.

### Bufalin inhibits diverse IHNV infection of EPC cells

**Fig 3:**
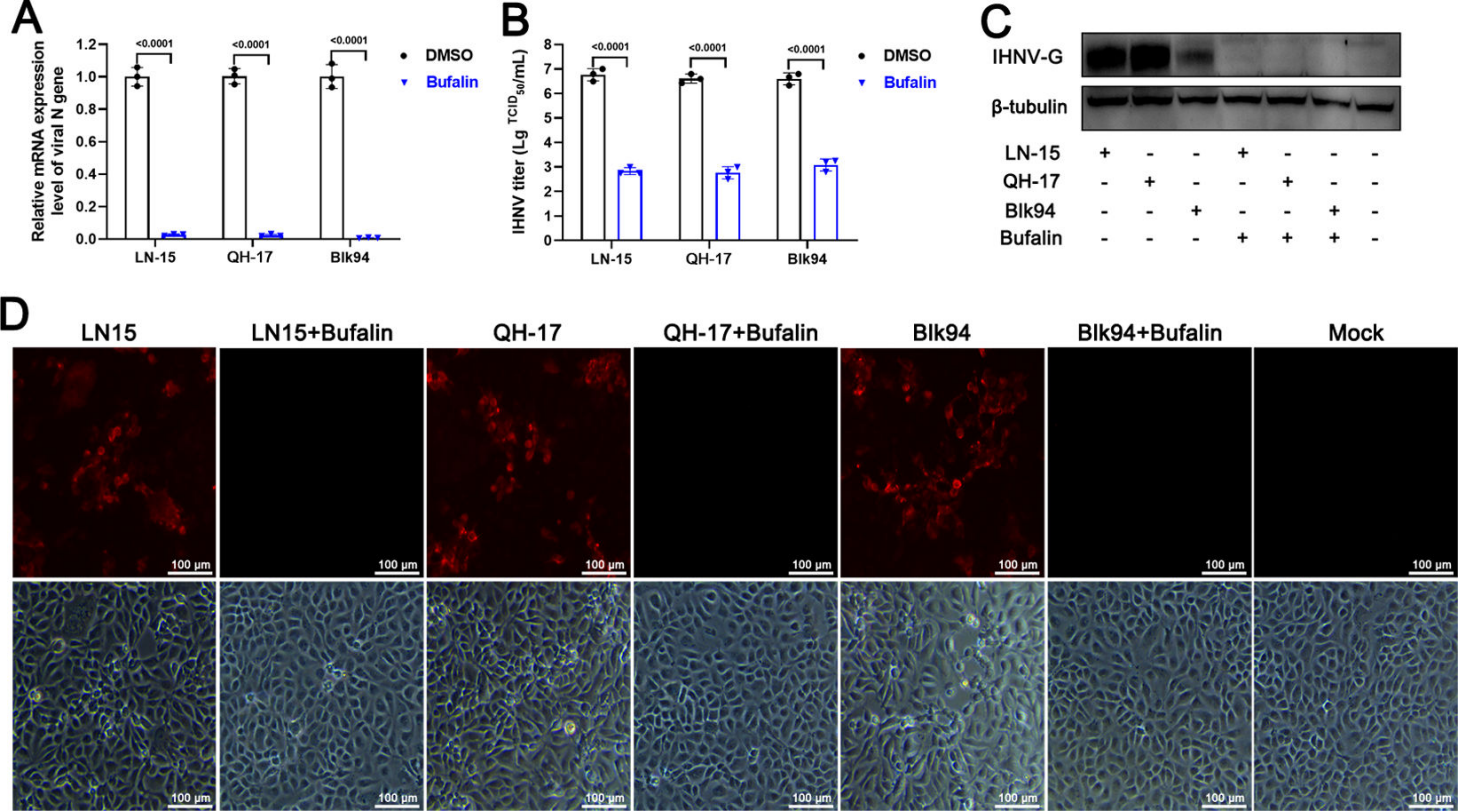
Antiviral effect of bufalin against infection of EPC cells with different strains of IHNV. (**A**) IHNV mRNA expression after bufalin treatment. (**B**) IHNV strain titers after bufalin treatment. (**C**) Western blotting IHNV G protein. (**D**) IFA detection of IHNV-infected cells. Red fluorescence represents IHNV-infected EPC cells in the field. The light image showed all the EPC cells in the field.

The results above showed that bufalin inhibited IHNV-Sn1203 infection of EPC cells. We assessed the effect of bufalin treatment on infection by IHNV strains Blk94, LN-15, and QH-17 (Fig. 3). RT-qPCR showed that intracellular replication of all the strains was significantly inhibited by bufalin, and RNA expression was 152-fold lower for Blk94, 39-fold lower for LN-15, and 44-fold lower for QH-17 compared with the DMSO control group at 48 h (Fig. 3A). The extracellular viral titers were also significantly inhibited, and the viral titer was 3.5 log lower for Blk94, 3.9 log lower for LN-15, and 3.8 log lower for QH-17 compared with the DMSO control group at 48 h (Fig. 3B). Western blotting showed that the G protein expression of all three virus strains was inhibited by bufalin at 48 h (Fig. 3C). IFA showed that bufalin significantly reduced the number of cells infected by all three IHNV strains compared with mock-treated group (Fig. 3D). These results suggested that bufalin significantly inhibited the infection of EPC cells by all strains of IHNV, and the inhibitory effect was not specific for a single strain.

### Pre-, co-, and post-treatment with bufalin inhibit IHNV infection

**Fig 4:**
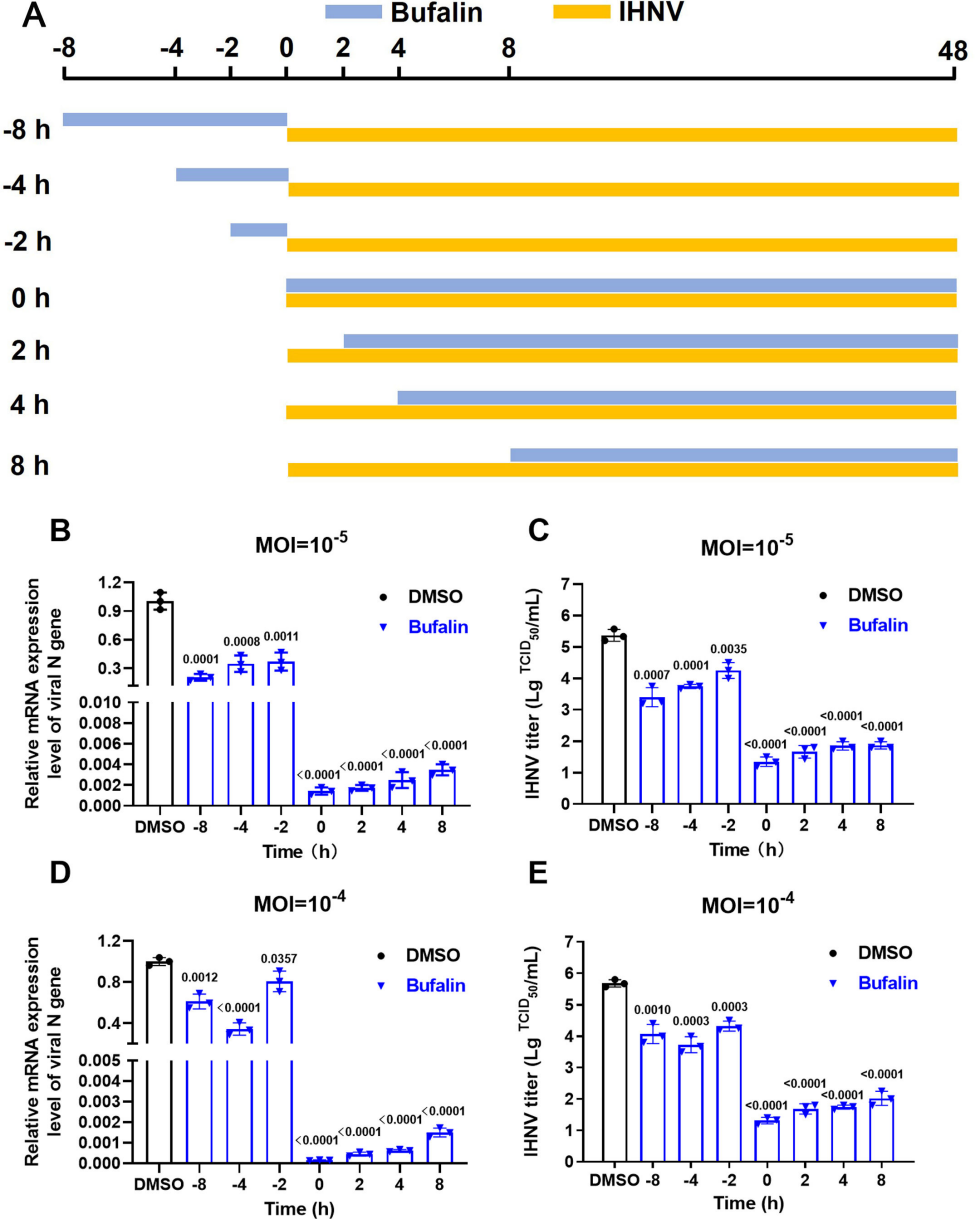
Effect of time of addition of bufalin on antiviral activity against IHNV. (**A**) Schematic diagram of time of addition. (**B**) IHNV mRNA expression after infection with IHNV MOI = 10^−5^ and bufalin treatment. (**C**) IHNV titer after infection with IHNV MOI = 10^−5^ infection and bufalin treatment. (**D**) IHNV mRNA expression after infection with IHNV MOI = 10^−4^ and bufalin treatment. (**E**) IHNV titer determination after infection with IHNV MOI = 10^−5^ and bufalin treatment.

To assess the stage at which bufalin works, we conducted a time of addition experiment (Fig. 4A). Time of IHNV infection was set to 0 h. For pretreatment, bufalin was added at −2, –4, and −8 h before IHNV infection. For co-treatment, bufalin was added at 0 h together with IHNV. For post-treatment, bufalin was added at 2, 4, and 8 h after IHNV infection. For all experiments, IHNV was added at 0 h and removed at 1 h (Fig. 4A). Pretreatment viral RNA expression was 4.85-fold lower at −8 h, 2.88-fold lower at −4 h, and 2.71-fold lower at −2 h compared with the control group at virus multiplicity of infection (MOI) = 10^−5^ (Fig. 4B). We detected viral titer and found that it was 1.96 log lower at −8 h, 1.62 log lower at −4 h, and 1.11 log lower at −2 h compared with the control group at MOI = 10^−5^ (Fig. 4C). These results suggested that pretreatment with bufalin significantly inhibited IHNV viral RNA (vRNA) expression, and the inhibition was more obvious with the prolongation of pretreatment. The co-treatment and post-treatment results showed that bufalin significantly inhibited vRNA expression level and extracellular viral titer, and these effects were stronger than after pretreatment. To verify these results, we performed a time of addition assay at a 10-fold higher MOI = 10^−4^ (Fig. 4D and E). The results were consistent with MOI = 10^−5^, and pretreatment, co-treatment, and post-treatment with bufalin all significantly inhibited IHNV infection.

### Bufalin inhibits IHNV attachment but not internalization

**Fig 5:**
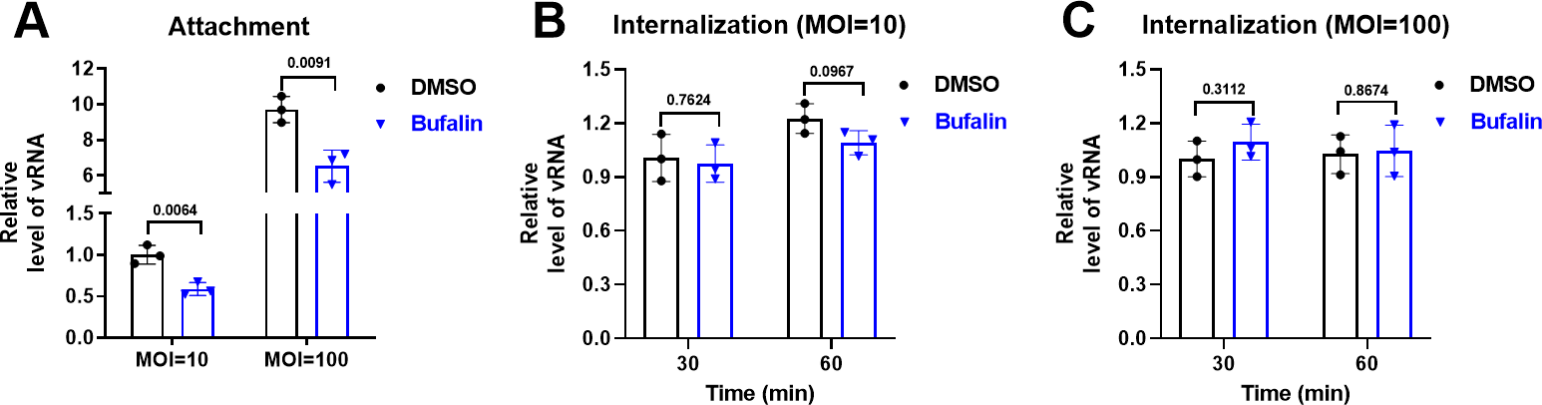
Antiviral effect of bufalin on IHNV attachment and internalization. (**A**) Antiviral effect of bufalin on IHNV attachment. (**B**) Antiviral effect of bufalin on IHNV internalization at MOI = 10. (**C**) Antiviral effect of bufalin on IHNV internalization at MOI = 100.

The time of addition results showed that pre-, co-, and post-treatment with bufalin all inhibit IHNV replication (Fig. 4B through E). IHNV undergoes attachment, internalization, and replication to achieve effective propagation. To further investigate at which stage of infection bufalin was effective, we measured IHNV vRNA expression after treatment with bufalin during IHNV attachment and internalization. To study viral attachment, IHNV and bufalin were added to EPC cells together and incubated at 4°C for 1 h to allow viral attachment to the cell surface. RT-qPCR showed that bufalin inhibited IHNV attachment at high and low MOI, and the inhibition efficiency was 41% at MOI = 10 and 33% at MOI = 100 (Fig. 5A). To study internalization, EPC cells were incubated with IHNV at 4°C for 1 h and incubated with bufalin at 15°C for 30 and 60 min. RT-qPCR showed that bufalin had no effect on IHNV internalization at low (10) (Fig. 5B) and high (100) MOI (Fig. 5C).

### Bufalin blocks viral RNA synthesis

**Fig 6:**
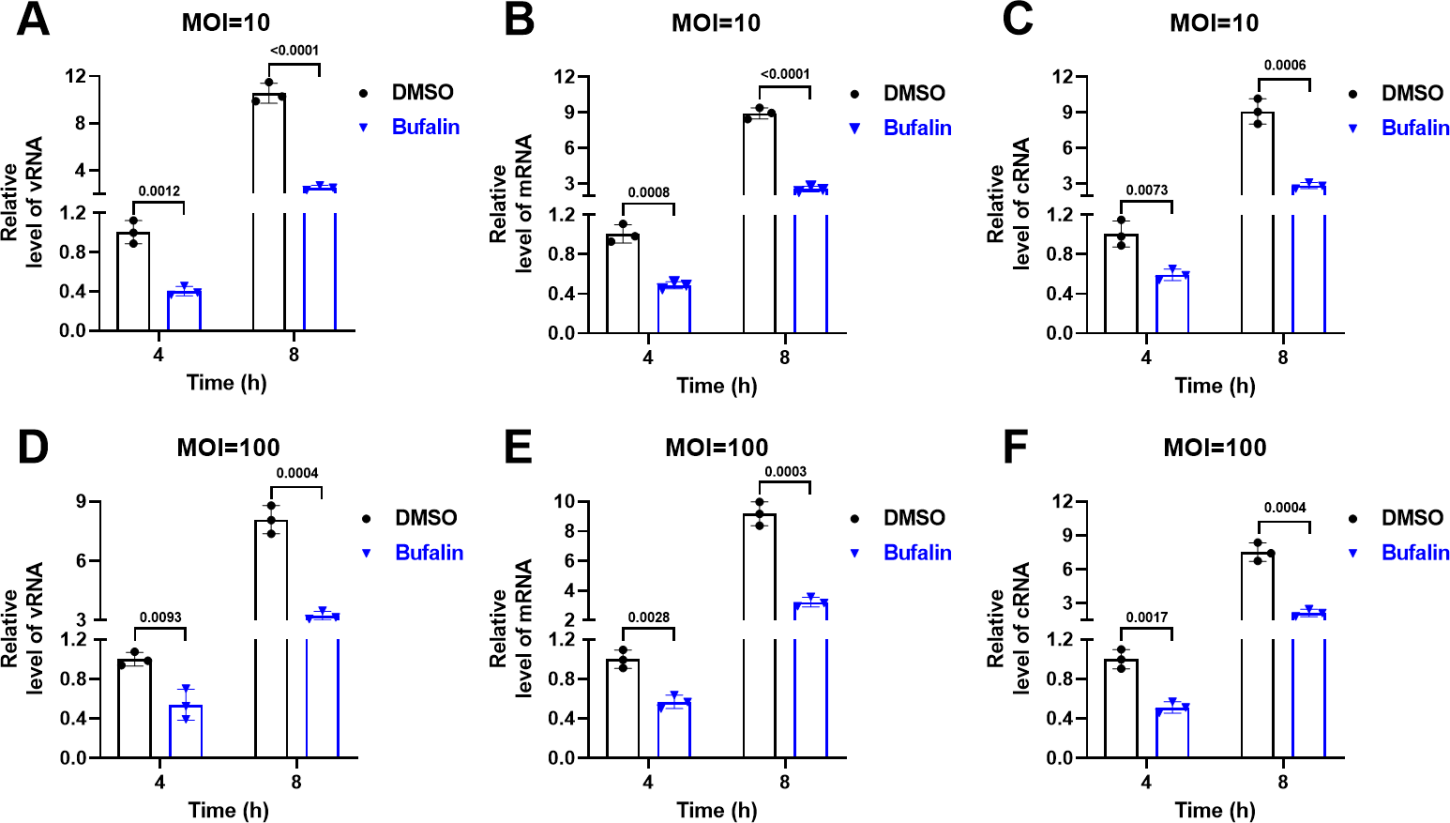
Effect of bufalin on IHNV RNA synthesis. Effect of bufalin on IHNV vRNA (**A**), mRNA (**B**), and cRNA (**C**) synthesis at MOI = 10. Effect of bufalin on IHNV vRNA (**D**), mRNA (**E**), and cRNA (**F**) synthesis at MOI = 100.

EPC cells were incubated with IHNV for 1 h. After culture at 15°C for 2 h, bufalin was added, and the cells were incubated for 4 and 8 h to assess the effect on viral replication. vRNA expression was significantly inhibited by bufalin: expression was 2.48-fold (MOI = 10) and 1.85-fold (MOI = 100) lower at 4 h and 4.17-fold (MOI = 10) and 2.5-fold (MOI = 100) lower at 8 h (Fig. 6A and D). Viral mRNA and complementary RNA (cRNA) expression was significantly inhibited by bufalin. mRNA expression was 2.06-fold (MOI = 10) and 1.75-fold (MOI = 100) lower at 4 h and 3.5-fold (MOI = 10) and 2.85-fold (MOI = 100) lower at 8 h (Fig. 6B and E). cRNA expression was 1.70-fold (MOI = 10) and 1.95-fold (MOI = 100) lower at 4 h and 3.21-fold (MOI = 10) and 3.50-fold (MOI = 100) lower at 8 h (Fig. 6C and F). These results suggested that bufalin blocked IHNV vRNA, mRNA, and cRNA synthesis.

### Bufalin inhibits IHNV infection *in vivo*

**Fig 7:**
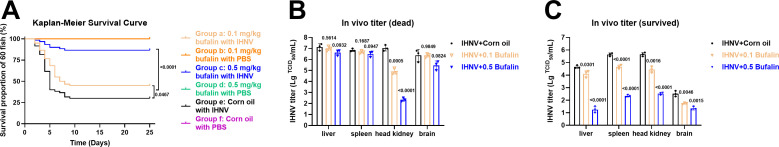
Antiviral effect of bufalin in rainbow trout. (**A**) Survival curve after bufalin treatment. (**B**) Viral load in different tissues of dead fish after bufalin treatment. (**C**) Viral load in different tissues of surviving fish after bufalin treatment.

The *in vitro* results showed that bufalin significantly inhibited IHNV infection of EPC cells and may be a promising drug against IHNV infection. To evaluate whether bufalin protects rainbow trout against IHNV infection *in vivo*, fish were treated with IHNV and different doses of bufalin. Rainbow trout treated with PBS (groups b, d, and f) did not show any mortality compared with the virus infection group (groups a, c, and e). The cumulative percentage mortality (CPM) of group e (corn oil with IHNV) was 70%, while both bufalin-treated groups showed lower CPM. Group a (0.1 mg/kg bufalin with IHNV) showed 55% CPM, which was significantly lower than that in group E (*P* = 0.0467). Although the relative percentage survival (RPS) of group a was 21.4%, 0.1 mg/kg bufalin delayed death caused by IHNV and reduced the mortality rate. Group c (0.5 mg/kg bufalin with IHNV) showed 11.7% CPM and 81% RPS, which differed significantly from group e (*P* < 0.0001) (Fig. 7A). To evaluate the protective effect of bufalin against IHNV infection *in vivo*, the dead and surviving rainbow trout were sampled to monitor viral load. Bufalin did not reduce IHNV load in the liver, spleen, and brain in expired fish, but viral load was significantly reduced in head kidney, and 0.1 mg/kg bufalin was 1.43-fold lower and 0.5 mg/kg bufalin was 3.00-fold lower than the corn oil mock-treated group (Fig. 7B). The viral load in survivors was significantly reduced in all bufalin-treated groups. The viral titers in the liver, spleen, head kidney, and brain in the corn oil mock-treated group were 1.13, 1.21, 1.28, and 1.32 log higher than those in the fish treated with 0.1 mg/kg bufalin (Fig. 7C). The viral titers in the liver, spleen, head kidney, and brain in the mock-treated group were 3.73, 2.41, 2.24, and 1.86 log higher than those in the fish treated with 0.5 mg/kg bufalin (Fig. 7C). These results suggested that bufalin significantly inhibited IHNV infection in rainbow trout.

### Bufalin inhibits IHNV infection via Na^+^/K^+^-ATPase

**Fig 8:**
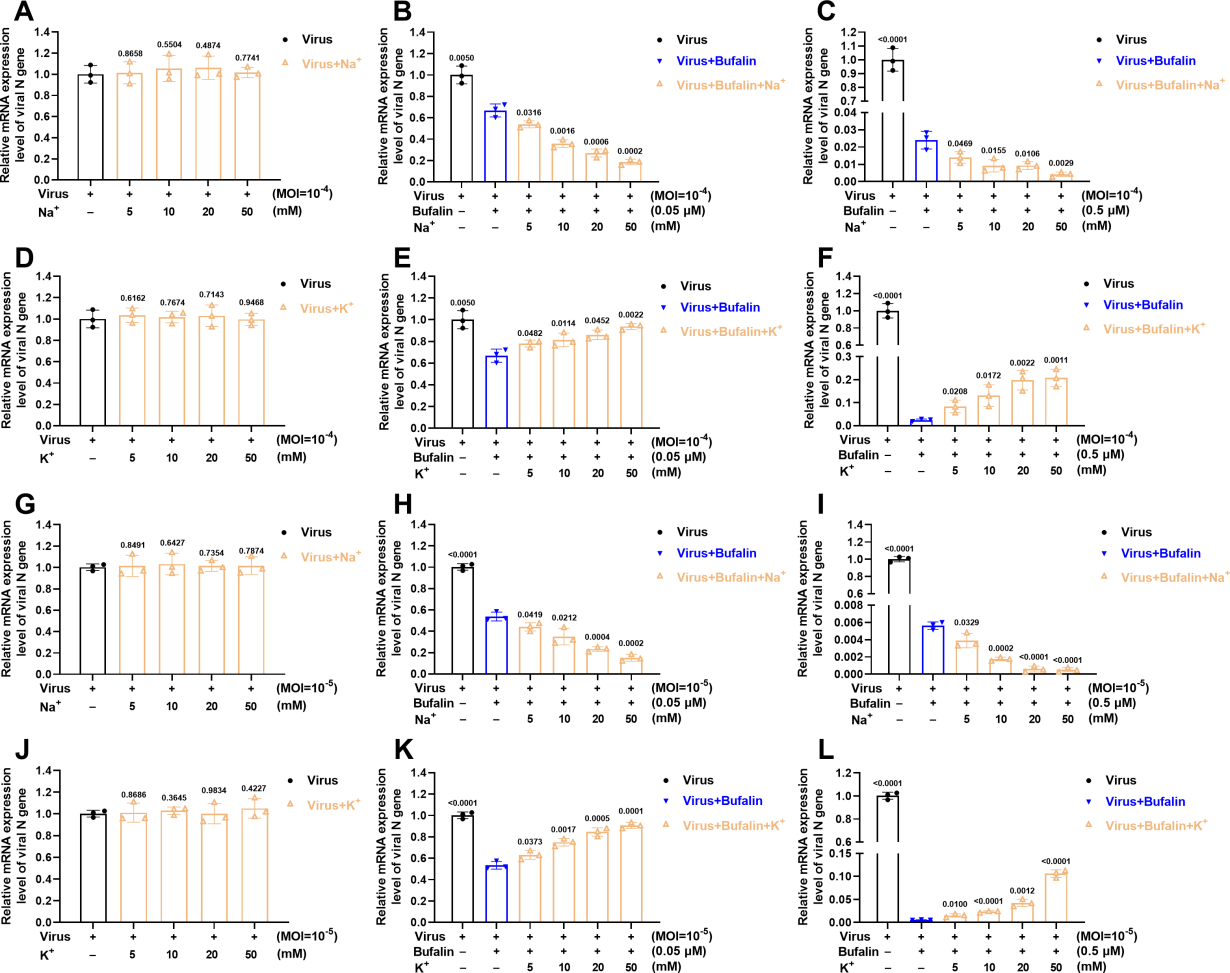
Identification of antiviral effect of bufalin via Na^+^/K^+^-ATPase. Viral mRNA expression levels with increasing concentration of Na^+^ coupled with different concentrations of bufalin at IHNV MOI = 10^−4^: without bufalin (**A**), 0.05 µΜ bufalin (**B**), and 0.5 µΜ bufalin (**C**). Viral mRNA expression levels with increasing concentration of K^+^ coupled with different concentrations of bufalin at IHNV MOI = 10^−4^: without bufalin (**D**), 0.05 µΜ bufalin (**E**), and 0.5 µΜ bufalin (**F**). Viral mRNA expression levels with increasing concentration of Na^+^ coupled with different concentrations of bufalin at IHNV MOI = 10^−5^, without bufalin (**G**), 0.05 µΜ bufalin (**H**), and 0.5 µΜ bufalin (**I**). Viral mRNA expression levels with increasing concentration of K^+^ coupled with different concentrations of bufalin at IHNV MOI = 10^−5^, without bufalin (**J**), 0.05 µΜ bufalin (**K**), and 0.5 µΜ bufalin (**L**).

Previous studies have reported that CGs can inhibit Zika virus and Chikungunya virus via Na^+^/K^+^-ATPase, which is the key target of CGs (33, 34). To determine whether the blockade of Na^+^/K^+^-ATPase was responsible for IHNV inhibition, EPC cells were infected with IHNV (MOI = 10^−4^ or 10^−5^) and treated with the indicated concentration of bufalin, Na^+^, and K^+^. Treatment with an increasing concentration of Na^+^ did not affect IHNV replication at high and low MOI (Fig. 8A and G). Treatment with bufalin at 0.05 and 0.5 µM inhibited IHNV replication (Fig. 8B and C), which was consistent with our results above (Fig. 1D). Combination with increasing concentration of Na^+^ increased the inhibitory effect of bufalin in a dose-dependent manner (Fig. 8B, C, H and I). Combination with increasing concentration of K^+^ also had no effect on IHNV replication at high and low MOI (Fig. 8D and J). Combination with increasing concentration of K^+^ alleviated the inhibitory effect of bufalin at 0.05 µM (Fig. 8E and K) and 0.5 µM (Fig. 8F and L), and the effect was proportional to the increasing K^+^ concentration. These results showed that the anti-IHNV effect of bufalin was proportional to extracellular Na^+^ concentration and inversely proportional to extracellular K^+^ concentration, and bufalin inhibited IHNV infection by targeting Na^+^/K^+^-ATPase.

## DISCUSSION

IHN is one of the main viral diseases that threatens the salmon and trout farming industry all over the world (1). IHNV is the pathogen of IHN for which outbreak often leads to great economic losses, and it has been a key problem that hampers the healthy development of salmon and trout farming (11). Currently, there is only one approved DNA vaccine for IHN worldwide (35). Although the DNA vaccine first approved in Canada has shown a good protective effect against IHNV infection, it still faces some biosafety problems. These issues included plasmid DNA inducing an autoimmune response and possible integration into the host genome. Hence, more effective vaccines and antiviral drugs are needed to prevent IHNV infection.

Traditional Chinese medicine is a promising resource for the development of antiviral drugs, and previous studies have shown good antiviral activity of such drugs against SARS-CoV-2 (36), herpes simplex virus type 1 (37), hepatitis B virus (38), respiratory syncytial virus (39), and influenza virus (40). To identify the effective antiviral candidates against IHNV, we screened 1,483 compounds from a traditional Chinese medicine monomer library. Bufalin exhibited antiviral activity against IHNV. Bufalin is one of the CGs first used for the treatment of cardiovascular diseases, but CGs have also shown antiviral activity in recent years (25–28). Although the antiviral activity of CGs has been studied against some viruses, the relationship between CGs and *Rhabdoviridae* has not been reported.

For the preliminary evaluation of the anti-IHNV effect of bufalin on EPC cells, CC_50_ was >20 µM (Fig. 1C) and IC_50_ was 0.1223 µM (Fig. 1D), and the drug SI was >163.5, which indicated the antiviral potential of bufalin. We also performed RT-qPCR, TCID_50_, western blotting, and IFA to confirm the effects of bufalin, and all the results showed that bufalin exhibited promising anti-IHNV activity (Fig. 2). We showed that bufalin also had antiviral activity against other IHNV strains, Blk94, LN-15, and QH-17, in EPC cells (Fig. 3), which confirmed that bufalin acted against all IHNV strains, rather than only one specific strain. Previous studies have shown that bufalin inhibited murine hepatitis virus, feline infectious peritonitis virus, MERS-CoV, and vesicular stomatitis virus infection prior to virus inoculation, but virus infection was not affected when bufalin was added after virus infection for 2 h (31). To further identify which stage that bufalin inhibited IHNV infection and whether bufalin exhibited the same antiviral mode as these viruses, we performed pre-, co-, and post-treatment with bufalin during IHNV infection. The results showed that pre-, co-, and post-treatment with bufalin all inhibited IHNV infection, and the inhibitory effect was increased with the duration of drug treatment (Fig. 4). To identify the exact stage at which bufalin inhibited IHNV infection, we divided early virus replication into three steps of attachment, internalization, and RNA replication. Bufalin significantly inhibited IHNV attachment to the cell surface, had no effect on viral internalization, and significantly inhibited IHNV RNA replication (mRNA, cRNA, and vRNA) (Fig. 5 and 6). These results suggest that bufalin inhibits IHNV infection through inhibiting viral attachment to cells and RNA replication, but the exact mechanism needs to be further investigated. The results of *in vitro* and *in vivo* studies are sometimes inconsistent. Although bufalin exhibited excellent anti-IHNV activity *in vitro*, the exact inhibitory effect still needs to be measured *in vivo*. We demonstrated that bufalin inhibited IHNV infection and significantly increased fish RPS (81% of 0.5 mg/kg bufalin) compared with mock-treated fish, which was confirmed by viral load monitoring (Fig. 7). These results suggest that bufalin may be a promising new drug against IHNV infection.

Previous studies have reported that the key target of CGs is Na^+^/K^+^-ATPase. For example, Zika and Chikungunya viruses are inhibited by CGs through action on Na^+^/K^+^-ATPase (33, 34). To further investigate the relationship between IHNV inhibition and Na^+^/K^+^-ATPase blockade, we infected EPC cells with IHNV with bufalin in combination with Na^+^ or K^+^. The inhibitory effect of bufalin increased with the concentration of Na^+^ and decreased with the increasing concentration of K^+^ (Fig. 8). These results showed that the anti-IHNV effect of bufalin was proportional to the extracellular Na^+^ concentration and inversely proportional to the extracellular K^+^ concentration, and bufalin inhibited IHNV infection by targeting Na^+^/K^+^-ATPase. The exact mechanism by which bufalin inhibits IHNV infection through Na^+^/K^+^-ATPase still needs to be investigated, and we proposed the following mechanism. Many intracellular biosynthetic and signaling pathways require ion transport. The inhibition of Na^+^/K^+^-ATPase disrupts ion transport that may change intracellular signal transduction, which in turn results in specific viral or cellular protein dysfunction, similar to that observed in alphavirus infection (41, 42). Further investigation of the mechanism by which blockade of Na^+^/K^+^-ATPase inhibits IHNV infection will clarify which host factors and pathways are required for IHNV infection. These factors can be used as additional drug targets to ameliorate IHNV disease.

In conclusion, 1,483 compounds from a traditional Chinese medicine monomer library were screened to obtain anti-IHNV drugs. The *in vitro* and *in vivo* studies all showed that bufalin significantly inhibited IHNV infection and may be a promising candidate drug against IHNV infection in rainbow trout.

## MATERIALS AND METHODS

### IHNV strains and cell lines

IHNV-Sn1203 strain (GenBank No: KC660147.1), LN-15 strain (GenBank No: MH170315.1), and QH-17 strain (GenBank No: MH170343.1) were isolated and stored in our laboratory (9, 43). IHNV-Blk94 strain (GenBank No: DQ164100), first described by Dr. Gael Kurath (44), was a gift by Dr. Hong Liu of the Shenzhen Entry-exit Inspection and Quarantine Bureau, China. The EPC cell line (CRL-2872, ATCC) was stored in our laboratory (43). EPC cells were cultured with MEM (8119087, Gibco, Shanghai, China) containing 1% penicillin–streptomycin (15070063, Gibco). For the conventional subculture of cells, the medium was supplemented with 10% fetal bovine serum (FBS) (2109290CP, Gibco) and 2% FBS for IHNV propagation.

### Compounds and screening assay

A traditional Chinese medicine monomer library (HY-L065) was purchased from MedChemExpress (Shanghai, China). The anti-IHNV drug screening was first performed by cell counting kit-8 (CCK8, B34304, Bimake, Shanghai, China) on EPC cells. EPC cells in 96-well plates were incubated with different drugs at 10 µM. After incubation for 6 h, the cells were infected with IHNV Sn1203 strain at an MOI = 0.1 for 1 h at 15°C and cultured with the drugs for 7 days. To monitor the antiviral activity, 10 µL CCK8 solution was added to each well, and the cells were incubated for 2 h at 15°C. The cell viability was monitored using a microplate reader at optical density (OD) 450 nm.

### Cytotoxicity and antiviral activity assay

EPC cells were seeded in 96-well plates and incubated with bufalin at 0.01, 0.02, 0.05, 0.1, 0.2, 0.5, 1, 2, 5, 10, and 20 µM. Cells treated with 0.1% DMSO were used as controls. After culture for 6 days, the cytotoxicity of bufalin was analyzed with the CCK8 kit, as described in the previous section. The concentration of bufalin that reduced OD_450_ to 50% of that of untreated cells was defined as CC_50_. For the antiviral activity assay, EPC cells in 96-well plates were treated with the same bufalin concentrations. After incubation for 6 h, the cells were infected with IHNV-Sn1203 strain at MOI = 0.1 for 1 h at 15°C. After culture for 6 days, the antiviral activity of bufalin was analyzed with the CCK8 kit using the previously described method. The inhibition rate was calculated as [(OD_450_ bufalin − OD_450_ virus control)/(OD_450_ control cell − OD_450_ virus control)] × 100%, and the IC_50_ of bufalin against IHNV was calculated using regression analysis. The SI for bufalin was defined as CC_50_/IC_50_.

### Antiviral effect of bufalin on different IHNV strains

EPC cells seeded in six-well plates were infected with different IHNV strains (Sn1203, LN-15, QH-17 or Blk94) for 1 h at MOI = 0.1, washed with PBS three times, and treated with 0.5 µM bufalin for 24 or 48 h. The antiviral effect of bufalin was assessed by RT-qPCR, viral titer determination, western blotting, and IFA. For RT-qPCR, the total RNA of cultured cells was extracted with the TRIzol reagent (10296028, Invitrogen, Carlsbad, CA, USA), and viral mRNA was analyzed using the primers listed in Table 1. For viral titer determination, cell culture supernatants were collected, 10-fold diluted, and added to EPC in 96-well plates. After culture for 7 days at 15°C, viral titers were determined by the Reed−Muench method. For western blotting, cells were collected using lysis buffer, and protein expression was analyzed using rabbit anti-IHNV-G polyclonal antibody (43), rabbit anti-β-tubulin monoclonal antibody (ab179513, Abcam, Cambridge, UK) as primary antibody, and HRP-labeled goat anti-rabbit IgG antibody (ab6721, Abcam) as secondary antibody. For IFA, cells were fixed with 4% paraformaldehyde and permeabilized with Triton X-100. The anti-IHNV-G antibody was used as the primary antibody, and Cy3-tagged goat anti-rabbit IgG antibody (ab97075, Abcam) was used as the secondary antibody.

**TABLE 1:** Primers used for IHNV RNA determination

Primer name	Sequence (5′−3′)	Target RNA
vRNA F	GGCCGTCATGGTGGCGAATTCCACGTCATACTTCGTCAGTAGAGC	vRNA
vRNA R	CCACTTTCGCAGATCCCAACAACA	
vRNA tag	GGCCGTCATGGTGGCGAAT	
cRNA F	GAGACCAGACCCGGACGAGGAGGA	cRNA
cRNA R	GCTAGCTTCAGCTAGGCATCTCTATCTGGGAGTCAGGTGGGGGTG	
cRNA tag	GCTAGCTTCAGCTAGGCATC	
mRNA F	GAGACCAGACCCGGACGAGGAGGA	mRNA
mRNA R	CCAGATCGTTCGAGTCGTTTTTTTTTTTTTTTTTTCAGTGGAATGA	
mRNA tag	CCAGATCGTTCGAGTCGT	
β-Actin F	GCCGGCCGCGACCTCACAGACTAC	β-Actin
β-Actin R	CGGCCGTGGTGGTGAAGCTGTAAC	

### Time of addition assay

EPC cells were seeded in six-well plates and infected with IHNV at MOI = 10^−4^ or 10^−5^. The timepoint when IHNV was inoculated into EPC cells was set as 0 h, and bufalin (0.5 µM) was added at −8, –4, −2, 0, 2, 4, and 8 h. After IHNV infection for 48 h, the supernatant and cells were collected. The supernatant was used to determine virus titer using the Reed−Muench method, while cells were lysed using TRIzol to obtain total RNA, and viral mRNA expression was determined by RT-qPCR. Cells incubated with DMSO were used as a negative drug control.

### Inhibitory efficiency of bufalin at different stages of IHNV infection

EPC cells were seeded in six-well plates 24 h before IHNV infection (MOI = 10 and 100). For the viral attachment assay, cells were co-treated with bufalin (0.5 µM) and IHNV at 4°C for 1 h and then collected after washing three times with PBS. For the viral internalization assay, cells were infected with IHNV at 4°C for 1 h and washed three times with PBS. The cells were treated with bufalin (0.5 µM) for 30 and 60 min and collected after washing with citrate buffer. For the RNA replication assay, cells were infected with IHNV for 1 h at 15°C and washed three times with PBS. After IHNV infection for 2 h, bufalin (0.5 µM) was added, and cells were collected after incubation for 4 and 8 h. IHNV vRNA, cRNA, and mRNA expression levels were measured by RT-qPCR with the primers listed in Table 1 to evaluate the inhibitory efficiency of bufalin.

### Bufalin inhibition of Na^+^/K^+^ ATPase

EPC cells were seeded in 12-well plates and infected with IHNV (MOI = 10^−4^ or 10^−5^) for 1 h. After washing with PBS, the cells were treated with bufalin (0.05 or 0.5 µM) in the presence of increasing concentrations of K^+^ (0, 5, 10, 20, or 50 mM) or Na^+^ (0, 5, 10, 20, or 50 mM). After IHNV infection for 48 h, the cells were collected, and the mRNA expression of IHNV was measured by RT-qPCR.

### Effect of bufalin against IHNV *in vivo*

Rainbow trout were bought from a local farm that had no outbreak of IHNV in the past 5 years, and the fish were tested to rule out carriage of IHNV. To test the effect of bufalin against IHNV *in vivo*, rainbow trout with a mean weight of 10 ± 2 g were divided into six groups. Groups a and b were injected intraperitoneally with 0.1 mg/kg bufalin, groups c and d were injected intraperitoneally with 0.5 mg/kg bufalin, and groups e and f were injected intraperitoneally with the same volume of corn oil. Groups a, c, and e were challenged with IHNV by intraperitoneal injection at a dose of 10^2^ pfu for each fish, and groups b, d, and f were challenged with the same volume of PBS by intraperitoneal injection. The mortality of these six groups was recorded daily for 25 days, and the efficacy of bufalin against IHNV was determined by CPM.

### *In vivo* viral load determination

Five rainbow trout of each group at each timepoint were sampled, and virus-targeted tissues were collected to monitor viral load. The liver, spleen, head kidney, and brain were collected, and 0.1 g tissue was ground with 500 µL PBS. After centrifugation, the supernatant was sterilized using a 0.22-µm sterile filter and then 10-fold gradient-diluted with MEM containing 2% FBS. The dilutions were added to 96-well plates containing EPC cells and cultured at 15°C, and the viral titer was calculated after 7 days.

### Statistical analysis

All the statistical analyses were performed using GraphPad Prism software (version 6.0), and the data are presented as means ± standard deviations. Student’s *t*-test was used to determine the differences between two groups, and values of *P* < 0.05 were considered to indicate a statistically significant difference.
